# Predicting donor, recipient and graft survival in living donor kidney transplantation to inform pretransplant counselling: the donor and recipient linked iPREDICTLIVING tool – a retrospective study

**DOI:** 10.1111/tri.13580

**Published:** 2020-02-24

**Authors:** Maria C. Haller, Christine Wallisch, Geir Mjøen, Hallvard Holdaas, Daniela Dunkler, Georg Heinze, Rainer Oberbauer

**Affiliations:** ^1^ Section for Clinical Biometrics Center for Medical Statistics, Informatics and Intelligent Systems (CeMSIIS) Medical University of Vienna Vienna Austria; ^2^ Nephrology Ordensklinikum Linz, Elisabethinen Linz Austria; ^3^ Department of Transplant Medicine Oslo University Hospital Oslo Norway; ^4^ Division of Nephrology and Dialysis Department of Medicine III Medical University of Vienna Vienna Austria

**Keywords:** donor survival, graft survival, kidney transplant, living donor, recipient survival, risk prediction, risks score, transplant counselling

## Abstract

Although separate prediction models for donors and recipients were previously published, we identified a need to predict outcomes of donor/recipient simultaneously, as they are clearly not independent of each other. We used characteristics from transplantations performed at the Oslo University Hospital from 1854 live donors and from 837 recipients of a live donor kidney transplant to derive Cox models for predicting donor mortality up to 20 years, and recipient death, and graft loss up to 10 years. The models were developed using the multivariable fractional polynomials algorithm optimizing Akaike’s information criterion, and optimism‐corrected performance was assessed. Age, year of donation, smoking status, cholesterol and creatinine were selected to predict donor mortality (*C*‐statistic of 0.81). Linear predictors for donor mortality served as summary of donor prognosis in recipient models. Age, sex, year of transplantation, dialysis vintage, primary renal disease, cerebrovascular disease, peripheral vascular disease and HLA mismatch were selected to predict recipient mortality (*C*‐statistic of 0.77). Age, dialysis vintage, linear predictor of donor mortality, HLA mismatch, peripheral vascular disease and heart disease were selected to predict graft loss (*C*‐statistic of 0.66). Our prediction models inform decision‐making at the time of transplant counselling and are implemented as online calculators.

## Introduction

Kidney transplantation is a cost‐effective treatment option for eligible end stage renal disease patients, because it is associated with improved survival, better quality of life and less costs compared to maintenance dialysis 1. Furthermore, current recommendations advocate kidney transplantation in a timely manner, at best performed preemptively to even avoid initiation of dialysis treatment 2. Given the organ shortage from deceased donors, a timed preemptive transplantation is usually only feasible with a living donor available. This entails additional benefits owing to the higher organ quality and shorter ischemia time compared to deceased donor kidney transplantation. It results in superior graft survival compared to deceased donor kidney transplants 3.

The decision‐making process for a living donor kidney transplantation, however, is complex and requires a careful assessment of the risks and benefits for both, the donor and the recipient. In particular, as the donor does not derive any medical benefit from a kidney donation, it is important to achieve a balance between preventing harm to the donor and improving the recipient’s prognosis. This risk‐benefit assessment rarely is straightforward because it largely depends—besides established clinical determinates of suitability to donate or receive a kidney—on how donor and recipient weigh their individual risks against the potential benefits; for example, a parent kidney donor is likely to accept a higher risk for oneself if the child’s prognosis is improved, even at a lower chance 4, 5, 6. Educating candidate donor and recipient pairs about potential adverse consequences is essential in order to inform decision‐making. Ideally, a valid risk stratification tool helps to quantify the risk for the donor and the benefits for the recipient tailored to the actual individual risk factors of each donor and recipient pair 7.

Although separate risk prediction models for donors and recipients were published in the past, we were unable to find a risk calculator to predict outcomes of donor/recipient pairs which clearly are not independent of each other. Two models were developed to predict recipient death at five years after transplantation, constituting a rather limited time horizon given the good long‐term prognosis expected from a living donor kidney transplant nowadays 8, 9. In addition, neither of the aforementioned models included risk factors arising from the donor or a summary estimate of the donor’s risk profile, while it is reasonable to assume a transmission of risks inherent in the donor to the recipient’s prognosis.

Thus, we identified a need for linked risk prediction models that predict relevant hard long‐term outcomes for the donor and recipient simultaneously to advance the evidence base for transplant counselling. The primary aim of this study therefore was to develop the donor and recipient linked iPREDICTLIVING tool to predict donor, recipient and graft survival following kidney donation or living donor kidney transplantation simultaneously taking a potential transmission of the donor’s risk profile to the recipient’s risk into account.

## Materials and methods

This manuscript is written following recommendations of the Transparent Reporting of a multivariable prediction model for Individual Prognosis Or Diagnosis (TRIPOD) statement for prediction studies 10.

### Study design and population

A retrospective cohort of first single‐organ living donor kidney transplant recipients and living kidney donors from the Oslo University Hospital, Oslo, Norway, were used to develop the risk prediction models. In Norway, a single centre, the aforementioned Oslo University Hospital, performs all kidney transplantations and 30–40% of all kidney transplants come from a living donor. Eligibility for a living donor transplant is determined according to international guidelines 11, 12.

All donors recorded in the database who donated a kidney between 1 January 1980 and 31 December 2007 were included in this analysis. As collection of recipient comorbidities commenced on 1 January 1995, all consecutive adult (age ≥18 years at initiation of renal replacement therapy) recipients of a living donor kidney between 1 January 1995 until 31 December 2007 were included in this analysis. Information on the degree of sensitization to donor specific HLA epitopes of the recipient is not available from this database, as it was not performed routinely before 2010 13.

### Definition of predictors and outcome variables

Outcomes of interest were donor death, recipient death and graft loss. Donor and recipient death were defined as all‐cause mortality, including death with a functioning graft for recipients. Donor and recipient survival time was defined as the time from kidney donation or transplantation until death, end of follow‐up, or 10 March 2015, whichever occurred first. Graft survival time was defined as the time from transplantation until either permanent return to dialysis treatment or second transplantation, counting death and end of follow‐up as censored observations.

We used donor and recipient characteristics available at the time of transplant counselling as candidate predictors to develop the risk prediction models.

### Prediction models

We developed three prediction models within the iPREDICTLIVING tool to predict: (i) donor death following living kidney donation (donor mortality model), (ii) recipient death following living donor kidney transplantation (recipient mortality model) and (iii) graft loss following living donor kidney transplantation (graft loss model).

Candidate predictors were determined based on clinical judgment and existing background knowledge on relevance of risk factors for donor and recipient outcomes. Candidate predictors for the donor death model were the following donor characteristics: age, body mass index, sex, smoking status, serum creatinine, fasting glucose, fasting total cholesterol, systolic and diastolic blood pressure, first degree relationship (defined as either offspring, sibling or parent of the recipient), any genetic relationship with the recipient and calendar year of donation. Candidate predictors for both recipients models were primary renal disease, presence of panel reactive antibodies, sum of HLA mismatch in class II (DR), sum of HLA mismatch in class I (A, B), diabetes mellitus, heart disease, cerebrovascular disease, peripheral artery disease, recipient sex, recipient age, calendar year of transplantation, renal replacement therapy modality (coded as binary dummy variables to compare haemodialysis, peritoneal dialysis and preemptive transplantation), and donor prognosis. As our aim was to develop linked risk prediction models that account for a potential transmission of the donor’s risk profile to the recipient’s risk, we calculated the linear predictor for each donor for all included corresponding recipients using our donor mortality model. This individual linear predictor of donor mortality was then used as a metric candidate predictor for both recipient models. In order to keep the model parsimonious and as simple as possible for bedside use, we further decided a priori to include the duration of pretransplant dialysis treatment in the prediction model only if one of the three dummy variables for renal replacement therapy modality were selected into that model and if the *C*‐statistic of the respective model improved by adding pretransplant dialysis duration.

### Statistical analysis

Continuous variables are expressed by mean and standard deviation, categorical variables are presented by frequencies and percentages. Data availability was very good with recipient characteristics being completely available in the database for all patients. Missing donor variables were replaced by multiple imputation with chained equations 14, 15.

### Model development

Multivariable Cox regression models were applied to develop the prediction models. Predictors were selected using the multivariable fractional polynomial algorithm at a *P*‐value threshold of 0.157, corresponding to a selection criterion according to the Akaike’s information criterion, and to allow possible nonlinear associations of donor age, and donor body mass index with donor mortality as well as recipient age with recipient mortality and graft loss 16, 17. Schoenfeld residuals were calculated, and absence of a correlation with follow‐up time confirmed the validity of the underlying assumption of proportional hazards in all models. Nonlinear associations of HLA mismatch in both recipient models were further tested using forward selection at a *P*‐value of <0.01 for HLA mismatch using ordinal coding for increasing sum of class I and II antibodies separately. Biologically plausible interactions of selected main effects were tested as recommended by Royston and Sauerbrei using forward selection with a *P*‐value of 0.01 for all two‐way interactions between age and sex with all other selected predictors 17.

### Model validation

We used 1000 bootstrap resamples for internal validation to calculate optimism‐corrected performance measures and to estimate global shrinkage 18. Prediction models were corrected for optimism by multiplying all coefficients in the model with the global shrinkage factor 19. Performance and validity of prediction models were assessed by (i) explained variation, the proportion of variability in the outcome that is explained by the model, (ii) discrimination, the ability of prediction models to separate individuals with different outcomes, using Uno's concordance statistic (*C*‐statistic) for 10‐year risk, and (iii) calibration, the agreement between observed and predicted risks, using visual inspection of calibration plots for 10‐year risk, and additionally 20‐year risk for donor death 20, 21, 22, 23. An optimism‐corrected *C*‐statistic was calculated according to Steyerberg 18. Calibration was first assessed for each bootstrap replication as follows. We calculated predicted probabilities for each individual by applying each bootstrap model to all subjects of the original data set. We categorized the predicted probabilities by their quintiles and computed the mean predicted probabilities and the Kaplan–Meier estimates of observed cumulative incidence in each quintile group. Finally, we averaged these quantities over the bootstrap replications and plotted the quintile‐group‐specific aggregated observed versus predicted risk.

Analyses were done with r software (R version 3.3.1) 24. The study was approved by the Ethics Committee of the Helse Sør øst (2009/1588) and performed in accordance with the Declaration of Helsinki and Istanbul.

## Results

**Figure 1:**
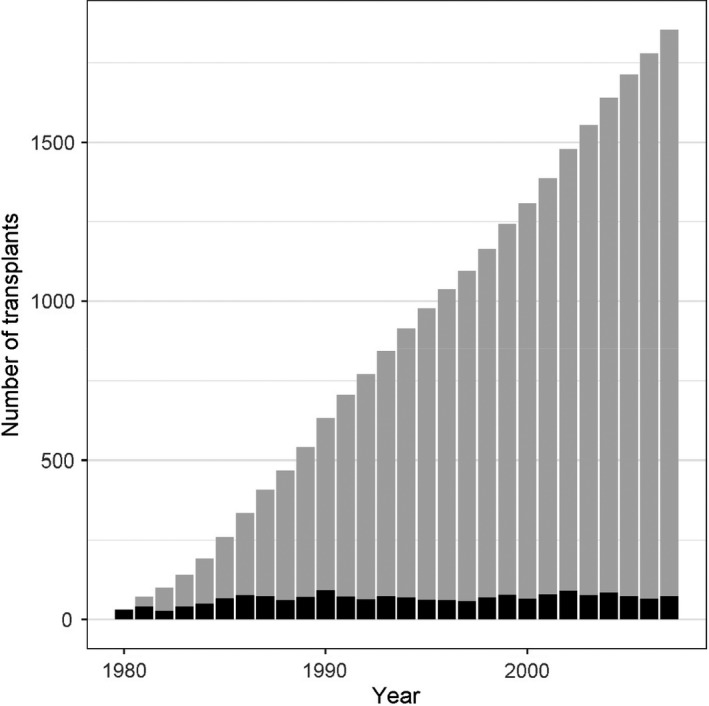
This bar chart shows the cumulative number of kidney transplants from live donor kidneys (= number of live donor kidney donations) per year from 1980 to 2007 that were included in this risk prediction modelling study. The black bars represent the number of new kidney donations/transplants each year, and the grey bars represent the cumulative sum of kidney donations/transplant until the respective year.

We identified 1854 donors and 837 recipients with a median follow‐up of 14.6 years for donors and 13.1 for recipients (Fig. 1). 195 (11%) donors and 255 (31%) recipients died, and 162 (20%) recipients experienced graft loss during the observation period until March 2015. Baseline characteristics of donors and recipients available at transplant counselling are given in Table 1.

**Table 1:** (a) Donor baseline characteristics available at transplant counselling. (b) Recipient baseline characteristics available at transplant counselling.

Donor, *n* = 1854	% Missing	Before donation
(a)		
Age (years; mean ± SD)	0	48.1 ± 12.5
Male donors (*n*, %)	0.3%	761 (41.2%)
BMI (mean ± SD)	14.6%	24.9 ± 3.3
Smoker (*n*, %)	21.4%	581 (39.9%)
Fasting glucose (mg/dl, mean ± SD)	21.4%	50.8 ± 5.8
Total cholesterol (mmol/l, mean ± SD)	15.4%	57.8 ± 12.9
Systolic BP (mmHg, mean ± SD)	2.6%	125.2 ± 11.5
Diastolic BP (mmHg, mean ± SD)	2.6%	78.3 ± 7.8

Recipient	*n* = 837
(b)	
Age (years; mean ± SD)	47.2 ± 14.5
Male recipients (*n*, %)	549 (65.6%)
First degree relation to donor (*n*, %)	608 (72.6%)
Diabetes mellitus (*n*, %)	102 (12.2%)
Cardiovascular disease (*n*, %)	80 (9.6%)
Primary renal disease	
Glomerulonephritis (*n*, %)	311 (37.1%)
Diabetic NP (*n*, %)	71 (8.5%)
Vascular NP (*n*, %)	95 (11.4%)
Sum of HLA MM (mean ± SD)	2.6 ± 1.6
Panel reactive antibodies (*n*, %)	25 (3.0%)
Calendar year of transplantation (median, IQR)	2002 (1998–2004)
Renal replacement therapy modality	
Preemptive transplantation (*n*, %)	303 (36.2%)
Haemodialysis (*n*, %)	417 (49.8%)
Peritoneal dialysis (*n*, %)	117 (14.0%)

### Donor mortality model

**Figure 2:**
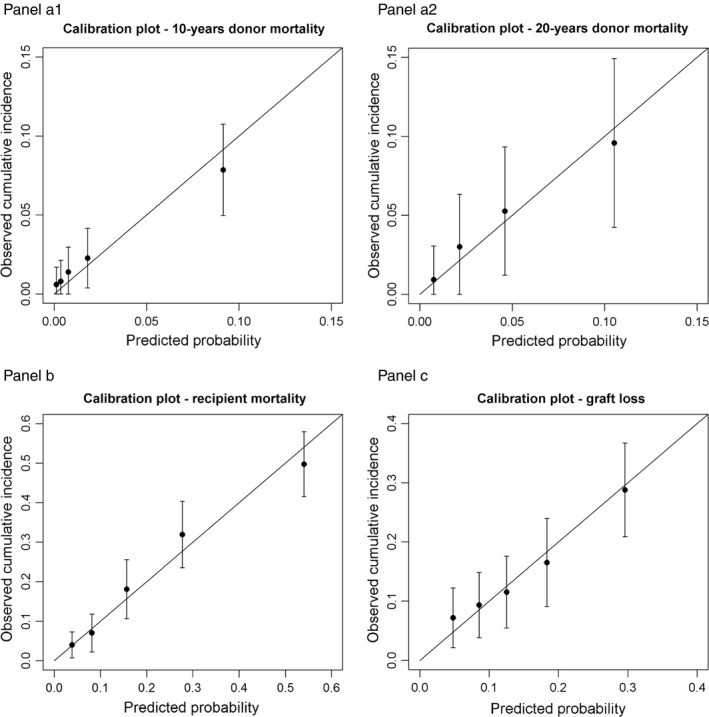
Calibration plots of all models (Panel a1 donor mortality model at 10, Panel a2 donor mortality at 20 years, Panel b recipient mortality model, Panel c graft loss model). The risk comparison between observed and predicted risk is grouped by quartiles of the predicted risk estimated by the models at ten and twenty years. 95% confidence intervals are added for the observed risks. Perfect agreement between observation and prediction is expressed by all dots lying on the diagonal. 95% confidence intervals intersecting the diagonal depict reasonable calibration of the model. Panel a1: calibration plot for donor mortality model at 10 years. Panel a2: calibration plot for donor mortality model at 20 years. Panel b: calibration plot for recipient mortality model. Panel c: calibration plot for graft loss model.

Age, calendar year of donation, smoking status, fasting serum cholesterol and serum creatinine were selected as predictors of donor mortality. Ten‐year baseline survival (at mean values of predictors) was 0.99, and 20‐year baseline survival was 0.95. The full prediction model with individual coefficients for all predictors is listed in Table 2a. Nonlinear associations between donor age or body mass index and mortality were not detected. With a global shrinkage factor of 0.97, the optimism‐corrected model exhibited an explained variation of 48.1%, and a *C*‐statistic of 0.81 (Table 3). The calibration plots of prediction of donor death within 10 and 20 years revealed reliable agreement between observed and predicted risks for donor mortality as the 95% confidence intervals of the predicted risks cover the diagonal representing perfect calibration (Fig. 2, panel a1 and a2). No interaction terms with age and sex were selected by the modelling algorithm.

**Table 2:** The equation parameters of each prediction model to estimate the 10‐year risk of donor mortality (a), recipient mortality (b) and graft loss (c).

(a)
**Donor mortality model**
The hypothetical donor profile (the ‘Individual Example Value’ column) assumes a 48‐year‐old donor candidate who is a nonsmoker, with a total cholesterol of 6.5 mmol/l, and a serum creatinine of 71 µmol/l. Calculation of the 10‐ and 20‐year risk estimate for donor mortality given this hypothetical donor can be done following the three steps as described above: First and second step: Compute the individual linear predictor by (0.1120 × 48) + (0.43 × 0) + (−0.1078 × 6.5) + (0.0182 × 71) = 5.9664. Third step for 10 year risk: $1\;-\;0.9921402^{exp\left(5.9664-7.2449\right)}$ and results in a calculated 0.22% risk of donor death within 10 years. Third step for 20 year risk: $1\;-\;0.9534074^{\exp\left(5.9664-7.2449\right)}$ and results in a calculated 1.3% risk of donor death within 20 years

(a) Predictor	Coefficient	Individual example value	Coefficient × individual example value	Range for continuous predictors
Donor age (years)	0.1120	48	5.3752	19–82
Current smoking status Smoker = 1 vs. non‐smoker = 0	0.4300	0	0	–
Total cholesterol (mmol/l)	−0.1078	6.5	−0.7008	1.5–12.5
Serum creatinine (µmol/l)	0.0182	71	1.2920	35–160
Individual linear predictor			5.9664	
Mean linear predictor			7.2449	
Baseline survival for 10‐year prediction			0.9921402	
Estimated 10‐year mortality risk			**0.22%**	
Baseline survival for 20‐year prediction			0.9534074	
Estimated 20‐year mortality risk			**1.3%**	

(b)
**Recipient mortality model**
The hypothetical recipient profile (the ‘Individual Example Value’ column) assumes a 60‐year‐old male transplant candidate who is transplanted preemptively or on peritoneal dialysis, with a glomerulonephritis as primary renal disease, with no previous cerebrovascular or peripheral arterial disease, and a full match in class II HLA antibodies (DR) with his potential living donor. Calculation of the 10‐year risk estimate for recipient mortality given this hypothetical recipient can be done following the three steps as described above: First and second step: Compute the individual linear predictor by (0.0663 × 60) + (0.3669 × 0) + (−0.6636 × 0) + (−0.7721 × 0) + (−0.8756 × 1) + (0.4280 × 0) × (0.3365 × 0) + (−0.1904 × 1) + (0.1451 × 0) = 2.9104. Third step: $1\;-\;0.8517^{\exp\left(2.9104-2.9416\right)}$ and results in a calculated 14.4% risk of recipient death within 10 years

(b) Predictor	Coefficient	Individual example value	Coefficient × value	Range for continuous predictors
Recipient age (years)	0.0663	60	3.9764	18–78
Dialysis vintage Hemodialysis = 1 vs peritoneal dialysis or preemptive transplantation = 0	0.3669	0	0	–
Primary renal disease* diabetic nephropathy = 0 vs else = 1	−0.6636	0	0	–
Primary renal disease diabetic nephropathy = 0 vs vascular nephropathy = 1	−0.7721	0	0	–
Primary renal disease diabetic nephropathy = 0 vs glomerulonephritis = 1	−0.8756	1	−0.8756	–
Cerebrovascular disease Yes = 1 vs no = 0	0.4280	0	0	–
Peripheral vascular disease Yes = 1 vs no = 0	0.3365	0	0	–
Recipient sex male = 1 vs female = 0	−0.1904	1	−0.1904	–
Sum of HLA mismatch in DR	0.1451	0	0	0–2
Individual linear predictor			2.9104	
Mean linear predictor			2.9416	
Baseline survival			0.8517	
Estimated 10‐year risk			**14.4%**	

(c)
**Graft loss model**
For the same hypothetical donor recipient pair, the 10‐year risk estimate for graft loss can be calculated assuming a sum of mismatch of HLA class I (AB) antibodies of 2, and no previous cardiovascular disease: First step: use the linear predictor of the donor (5.9664) as calculated in (a). Second step: Compute the individual linear predictor by (0.2656 × 5.9664) + (−0.0299 × 60) + (0.2245 × 0) + (0.6319 × 0) + (−0.1270 × 2) + (−0.7848 × 0) + (0.4925 × 0) = −0.4633. Third step: $1\;-\;0.8806^{\exp\left(-0.4633\;\text{to}\;0.8306\right)}$ and results in a calculated 3.4% risk of graft loss within 10 years

(c) Predictor	Coefficient	Individual example value	Coefficient × value	Range for continuous predictors
Linear predictor for donor (from a)	0.2656	5.9664	1.5846	
Recipient age (years)	−0.0299	60	−1.7938	18–78
Dialysis vintage Haemodialysis = 1 vs peritoneal dialysis or preemptive transplantation = 0	0.2245	0	0	–
Sum of HLA mismatch in DR	0.6319	0	0	0–2
Sum of HLA mismatch in A,B	−0.1270	2	−0.2541	0–4
Peripheral vascular disease Yes = 1 vs no = 0	−0.7848	0	0	–
Cardiovascular disease Yes = 1 vs no = 0	0.4925	0	0	–
Individual linear predictor			−0.4633	
Mean linear predictor			0.8306	
Baseline survival			0.8806	
Estimated 10‐year risk			**3.4%**	

Each table also includes a specific example of a hypothetical donor/recipient pair (the ‘Individual Example Value’) to illustrate the calculation procedure. Coefficients of all predictors, as well as the mean linear predictors, were multiplied with the appropriate shrinkage factor. Calendar year of donation/transplantation was fixed at the value for 2007, referring to the latest date of a donation/transplantation in the database, and was accounted for in the respective mean linear predictor. A donor/recipient profile can be inserted in the ‘Individual Example Value’ column. Calculation of the 10‐year risk estimate for the respective event given the inserted donor/recipient values can be done in three steps as follows: First, the individual example values are multiplied with the respective optimism‐corrected coefficients that were derived from the cox model equation and are provided in the column ‘Coefficient’. The column ‘Coefficient × Individual Example Value’ provides the results of this multiplication for an illustrative example in all tables. Second, the sum of the ‘Coefficient × Individual Example Value’ is then calculated for each individual to get the ‘Individual Linear Predictor’. Third, the estimated 10‐year risk of the respective event is then calculated as 1 minus the survival rate at 10 years (‘Baseline Survival’ in the table), raised to the power of the exponent of the ‘Individual Linear Predictor’ minus the ‘Mean Linear Predictor’ or, in equation form: $1\;-\;S_{10}^{exp(individual\;LP-mean\;LP)}$.

Bold values indicate calculated risks for the hypothetical donor and recipient pair.

* Primary renal disease is a categorical predictor with four groups, diabetic nephropathy, vascular nephropathy, glomerulonephritis and else. Diabetic nephropathy was used as reference group.

**Table 3:** Performance measures of prediction models.

Model	Performance measure		
	Optimism‐corrected *C*‐statistic	Explained variation (%)	Global shrinkage factor
Donor mortality model	0.81	48.1	0.97
Recipient mortality model	0.77	25.4	0.94
Graft loss model	0.66	10.2	0.88

### Recipient mortality model

Recipient age, recipient sex, calendar year of transplantation, renal replacement therapy modality, primary renal disease, cerebrovascular disease, peripheral artery disease, HLA class II mismatch were selected as predictors of recipient mortality (Table 2b). Nonlinear associations of recipient age and recipient mortality were not detected. Ten‐year baseline survival was 0.85, and the global shrinkage factor was 0.94. For recipient mortality, the optimism‐corrected *C*‐statistic was 0.77, and explained variation was 25.4% (Table 3). Calibration of the recipient mortality model showed reliable agreement between observed and predicted risks indicated by 95% confidence intervals covering the diagonal (Fig. 2, panel b). Recipient age and sex were not identified as effect modifiers of any other predictor in the model.

### Graft loss model

This model contained recipient age, pretransplant dialysis vintage, the summary score of donor prognosis, HLA class I and II mismatch, peripheral vascular disease, and heart disease, and yielded a 10‐year baseline survival for the graft of 0.88 (Table 2c). Model building procedures did not identify a nonlinear association of age and graft loss. For the prediction of graft loss, the model had a global shrinkage factor of 0.88, a *C*‐statistic of 0.66, and explained variation was 10.2% (Table 3). Agreement between observed and predicted risk of graft loss was reliable as indicated by the calibration plot (Fig. 2, panel c). There was no effect modification by age and sex.

### Online risk calculator

All three prediction models were implemented in the online tool iPREDICTLIVING and are available at http://www.meduniwien.ac.at/nephrogene/index.php/data/iPREDICTLIVING. The coefficients and baseline survival for calculating donor and recipient mortality as well as graft loss at 10 years in a three‐step calculus are provided in Table 2, along with an example based on a hypothetical donor and recipient pair.

## Discussion

Our iPREDICTLIVING tool is the first to provide linked risk prediction models for important long‐term outcomes for both, the donor candidate for as well as the recipient candidate of a living donor kidney transplantation allowing simultaneous risk estimation for the donor and recipient candidate at the time of transplant counselling.

Careful discussion of a donor candidate’s individual risks with individualized estimates of short‐ and long‐term risks is recommended 12, 25, but individualized prediction of donor outcomes was only available for end stage renal disease or impaired kidney function after donation so far 26, 27, 28. Despite several analyses of donor mortality in comparison with the general population, our study is the first to provide a bedside risk prediction tool using only variables available at the time of decision‐making to estimate a donor candidate’s risk of death at 10 years after kidney donation with excellent model performance 29, 30, 31, 32.

While donor safety must certainly be a prerequisite for a kidney donation, favourable outcomes for the recipient are pivotal at the same time to justify nephrectomy in a healthy individual. As a consequence donor and recipient outcomes need to be taken into account simultaneously when the benefits and harms of a live donor kidney transplantation are discussed in the process of informed decision‐making. Our analysis shows that linked risk prediction for the donor and recipient candidate is feasible using a summary measure of donor prognosis as candidate predictor for prediction of recipient outcomes. Two other prediction models for recipient mortality were previously published, but none considered donor characteristics making them less useful as an aid for educating potential donor and recipient pairs 8, 9.

A number of models for prediction of graft loss were published, but most of them apply to grafts from deceased donor kidney transplantation, or suffer from insufficient reporting or methodological flaws 33. In particular, models that include predictors that become available only *after* kidney transplantation are inapplicable for individualized prediction at the time of decision‐making *before* transplantation 34, 35. Two recent studies developed risk prediction models for graft loss including donor information, but both use data from the U.S. Scientific Registry of Transplant Recipients 36, 37. While this registry is clearly a large well‐maintained database, differences between healthcare systems and ethnicities in the United States and Europe might avert applicability of the aforementioned models outside the United States 38.

When using our models for individualized risk prediction, some limitations need to be considered. First of all, prediction models need to be interpreted in light of their natural limitation: a prediction can never predict whether an individual will have the event or not, but is a mathematical equation to quantify the chance of the event for a group of individuals with similar baseline characteristics. As such, prediction models can only explain the observed interindividual variation in outcomes to a certain extent, which can be quantified with the so‐called explained variation. For our donor mortality model, this performance measure is as high as 48%, while it is only 10% for the graft loss model can be explained with our model. Although this number seems small at first sight, it is reasonable given the many immune and nonimmune processes that may lead to graft loss, the majority of which develop after transplantation and as such past the time point of decision‐making. Besides this, a small double‐digit number is within the range of prediction models in other medical fields 39, 40. Unfortunately, we were unable to compare this performance measure to other frequently used prediction models or models in the field of kidney transplantation because it is not routinely reported 33, 41.

Despite the fact that we corrected prediction models and performance measures for optimism by internal validation using bootstrap resampling, we were not able to evaluate transportability to other populations by external validation due to lack of access to another cohort with similarly long and complete follow‐up. In general, such external validation, and if necessary, recalibration of prediction models to adopt to other populations and more contemporary eras should precede any application in clinical practice, but should preferably be done by independent research groups 42. However, we carefully followed international reporting guidelines for prediction studies, disclosing all necessary details of the prediction models to facilitate properly conducted independent external validation and recalibration studies. Our meticulous reporting highlights the solid methodology and distinguishes our work from other risk prediction studies that generally prevent their validation by their poor reporting 43. We provide a solid evidence base with fully and transparently reported risk prediction models that may be updated, recalibrated, and adopted to other cohorts, even in cohorts with shorter follow‐up or smaller sample sizes, as our sample size is sufficient to complement smaller data sets for updating our models 44.

Also, predicting recipient death and graft loss separately and therefore censoring for death in the model for graft loss may have introduced some concerns about our handling of competing risk. However, our rationale supporting this deliberate decision was grounded on the patient’s perspective who is primarily interested in graft loss as long as he or she is alive. We acknowledge that the risk to die for recipients may change after graft loss, and multistate models may be used to model transition hazards in order to simulate the impact of health policy changes on outcome event rates 45. Changing mortality hazards over time are also adequately dealt with by our direct prediction approach, which involved estimating the baseline hazard function non‐parametrically after the Cox model parameters were computed. We also want to be very clear that our prediction model for donor death is not an estimation of the impact of donation on donor survival as all individuals in the data set used for developing the iPREDICTLIVING tool had donated a kidney.

Not only in risk prediction studies, but in research, generally one needs to find a balance between the need of long‐term follow‐up and the need to use contemporary data to address potential era effects. We therefore decided to include donors from as early as 1980 for prediction of donor death, because we felt that there was only little change in practice over time and a long enough follow‐up after kidney donation is needed to facilitate observation of the rare event of death in these healthy individuals. Under similar considerations, we decided to include recipients for the models to predict recipient mortality and graft loss only after 1995, because more modern immunosuppression with tacrolimus and mycophenolate mofetil were not used before this date, and thus, this change in practice could have had a larger influence, while at the same time, a ten‐year observation period for recipient death and graft loss are reasonable and do constitute a longer follow‐up than in most transplant studies.

Our study has a number of strengths. First and foremost, the excellent quality of the data for donor and recipient characteristics available at transplant counselling as well as outcome data through follow‐up facilitated the development of linked risk prediction models in the first place. The database captures all living donor kidney transplants performed in Norway with no one lost to follow‐up. Besides the advantages of a well‐maintained national registry, we have diligently analysed the data, using state‐of‐the‐art methods to develop and validate risk prediction models including fractional polynomial functions for variable selection to determine the best linear or nonlinear form of continuous predictors and optimism correction of prediction as well as performance measures to provide trustworthy risk calculators for bedside use as previously shown 17, 18, 39. Furthermore, we implemented the models as online risk calculator, utilization of which is convenient requiring easy to obtain donor and recipient information and may assist in risk communication to support informed decision‐making during transplant counselling.

In conclusion, our donor and recipient linked IPREDICTLIVING tool perform well to predict donor mortality, recipient mortality and graft loss simultaneously for a donor and recipient candidate and can be used to inform decision‐making not only among an individual donor recipient candidate pair but also for paired kidney exchange programmes to further increase the benefits from live donor kidney transplantation in a wider pool of suitable donor and recipient pairing.

## Funding

Dr. Maria C. Haller was an ERBP research fellow until August 2017. European Renal Best Practice (ERBP) is the official guidance issuing body of the European Renal Association—European Dialysis and Transplant Association (ERA‐EDTA). Dr. R. Oberbauer was supported by the Vienna Science and Technology Fund (WWTF# LS16‐019) and the Austrian Science Fund (grant #P25726). The study received no direct funds.

## Authorship

MH: developed the study design, performed the study and wrote the manuscript. CW: performed the computations and wrote the manuscript. GM and HH: collected the data. DD and GH: verified the analytical methods. RO: perceived the original idea. GH and RO: supervised the study. All authors discussed the results, reviewed the manuscript, approved its final version and contributed important intellectual content.

## Conflict of interests

The authors of this manuscript have no conflict of interests related to this manuscript to disclose.
